# Influence of Martian regolith analogs on the activity and growth of methanogenic archaea, with special regard to long-term desiccation

**DOI:** 10.3389/fmicb.2015.00210

**Published:** 2015-03-20

**Authors:** Janosch Schirmack, Mashal Alawi, Dirk Wagner

**Affiliations:** ^1^Alfred Wegener Institute, Helmholtz Center for Polar and Marine Research - Research Unit Potsdam, PotsdamGermany; ^2^GFZ German Research Centre for Geosciences, Section 4.5 Geomicrobiology, PotsdamGermany

**Keywords:** methanogenic archaea, long-term desiccation, Martian regolith analogs, quantitative PCR, propidium monoazide, Mars

## Abstract

Methanogenic archaea have been studied as model organisms for possible life on Mars for several reasons: they can grow lithoautotrophically by using hydrogen and carbon dioxide as energy and carbon sources, respectively; they are anaerobes; and they evolved at a time when conditions on early Earth are believed to have looked similar to those of early Mars. As Mars is currently dry and cold and as water might be available only at certain time intervals, any organism living on this planet would need to cope with desiccation. On Earth there are several regions with low water availability as well, e.g., permafrost environments, desert soils, and salt pans. Here, we present the results of a set of experiments investigating the influence of different Martian regolith analogs (MRAs) on the metabolic activity and growth of three methanogenic strains exposed to culture conditions as well as long-term desiccation. In most cases, concentrations below 1 wt% of regolith in the media resulted in an increase of methane production rates, whereas higher concentrations decreased the rates, thus prolonging the lag phase. Further experiments showed that methanogenic archaea are capable of producing methane when incubated on a water-saturated sedimentary matrix of regolith lacking nutrients. Survival of methanogens under these conditions was analyzed with a 400 day desiccation experiment in the presence of regolith analogs. All tested strains of methanogens survived the desiccation period as it was determined through reincubation on fresh medium and via qPCR following propidium monoazide treatment to identify viable cells. The survival of long-term desiccation and the ability of active metabolism on water-saturated MRAs strengthens the possibility of methanogenic archaea or physiologically similar organisms to exist in environmental niches on Mars. The best results were achieved in presence of a phyllosilicate, which provides insights of possible positive effects in habitats on Earth as well.

## Introduction

The present day Mars is considered hostile to life as we know it on Earth. However, at the time when life first evolved on our planet, the environmental conditions might have been similar to those on early Mars (Carr, 1989, 1996; Durham et al., 1989; McKay and Davis, 1991; McKay et al., 1992). Therefore, it is possible that life might have simultaneously evolved on both planets. The detection of methane in the Martian atmosphere has been interpreted as a sign of possible biologic activity, amongst other interpretations (Formisano et al., 2004; Krasnopolsky et al., 2004; Mumma et al., 2009; Geminale et al., 2011); however, the latest measurements performed by a tunable laser spectrometer onboard the rover *Curiosity* indicated that the average methane concentration on Mars (at least in the Gale crater region) is approximately six times lower than what was originally estimated (Webster et al., 2013). Nevertheless, temporarily higher concentrations of methane could be observed with measurements conducted over a complete Martian year (Webster et al., 2015).

On Earth, the only biogenic source of methane is methanogenesis, and thus, methanogenic archaea are regarded as model organisms for possible life on Mars (Boston et al., 1992; Weiss et al., 2000; Jakosky et al., 2003; Morozova et al., 2007). Methanogenic archaea have evolved under early Earth conditions, and they are anaerobes that are capable of growing chemolithoautotrophically with hydrogen and carbon dioxide as sole energy and carbon sources, respectively. Although water might be available on the Martian surface-near subsurface (Möhlmann, 2010a,b; Möhlmann and Thomson, 2011), any possible life on Mars has to be able to withstand seasonal desiccation because Mars is considered a dry planet. Previous studies (Morozova et al., 2007) have shown the survival potential of methanogenic archaea – especially strains isolated from permafrost-affected soils such as *Methanosarcina soligelidi* SMA-21 (Wagner et al., 2013) – when exposed to simulated diurnal variations of Mars analog thermo-physical surface conditions, such as temperatures between –80 and +20°C, changing water activity between a_w_ 0 and 1, and a pressure of 6 mbar. Methanogenic archaea from permafrost environments also showed high resistance to freezing at –80°C, high salt concentrations up to 6 M NaCl (Morozova and Wagner, 2007) and methane production under simulated Mars subsurface conditions at a temperature of –5°C and pressure of 50 kPa (Schirmack et al., 2014a).

Because soil properties and the composition of the sedimentary matrix have a strong influence on the microbial activity and distribution on Earth (e.g., Görres et al., 2013; Rosa et al., 2014), the soil properties are most likely also a very important factor for the habitability of Mars. Therefore we investigated the influence of three different types of Martian regolith analogs (MRAs) on the growth and metabolic activity of three methanogenic strains from permafrost and non-permafrost environments. The regolith mixtures represent differently altered Martian soils, including sulfate-rich deposits and phyllosilicates, and have been designed according to soil types that can be found on Mars (Poulet et al., 2005; Chevrier and Mathé, 2007). The underlying hypothesis is that the properties of the regolith mixtures, due to their mineral composition, may affect the activity of methanogens. Other studies on methanogenic archaea from non-permafrost environments have shown inhibitory effects of Martian regolith analogs on methane production (Kral and Altheide, 2013).

Therefore, the aims of this study are to determine (i) the survival potential of methanogenic archaea from permafrost and non-permafrost environments under long-term desiccation (400 days) and (ii) the impact of components of different Martian regolith analogs (MRA) at increasing concentrations, with/without nutrient supplements, on the activity and growth of the methanogenic archaea. Survival was estimated via reincubation of the organisms in fresh medium and determination of the number of viable cells via propidium monoazide (PMA) treatment followed by quantitative PCR. The results of this study contribute to the understanding of factors influencing the survival rate of methanogens under extreme environmental conditions and to the understanding how methanogens were successful over the time from early Earth up to now, since the last common ancestor of all archaea might have been a methanogen (Gribaldo and Brochier-Armanet, 2006).

## Materials and Methods

### Organisms and Growth Media

Three strains of methanogenic archaea were used in these experiments: (i) *Methanosarcina soligelidi* SMA-21 isolated from the active layer of permafrost in the Lena Delta, Siberia (Wagner et al., 2013); (ii) *Methanosarcina mazei* DSM 2053^T^ (obtained from the Leibniz Institute DSMZ-German Collection of Microorganisms and Cell Cultures-DSMZ) isolated from a sewage sludge plant (Mah, 1980; Mah and Kuhn, 1984; Maestrojuán et al., 1992), which is the phylogenetically closest strain to *M. soligelidi* SMA-21; (iii) *Methanobacterium movilense* MC-20 (Schirmack et al., 2014b) isolated from the anoxic sediment of a subsurface thermal groundwater lake in the Movile Cave, Romania.

Two different anaerobic growth media were used to cultivate the organisms. *Methanosarcina soligelidi* SMA-21 and *Methanosarcina mazei* were incubated on MW medium (described in Schirmack et al., 2014a), and *Methanobacterium movilense* MC-20 was incubated on MB medium (described in Schirmack et al., 2014b). All strains were incubated in sealed, 125-ml serum bottles containing 50 ml of medium, and the headspace was filled with a gas mixture of 100 kPa H_2_/CO_2_ (80:20 v/v) and 200 kPa overpressurization with N_2_/CO_2_ (80:20 v/v). All incubations were at 28°C and in the dark but without shaking.

During the course of the experiments, a 300-μl sample was taken from the headspace at time intervals to check for methane production by gas chromatography (GC) using the GC 6890 from Agilent Technologies equipped with a capillary column Plot Q (length 15 m, diameter 530 μm) and a flame ionization detector (FID). Cell numbers were estimated through counting in a Thoma chamber with a Zeiss Axioscop 2 microscope (Carl Zeiss, Germany).

### Martian Regolith Analogs (MRAs)

Three different types of MRAs were used in this study. The first, JSC Mars-1A, was obtained from Orbital Technologies Corporation (Madison, WI, USA). JSC Mars-1A is a palagonitic tephra (volcanic ash altered at low temperatures) that was mined from a cinder quarry and sieved to the <1 mm fraction. The elemental composition is reported in **Tables 1** and **2**.

**Table 1:** Mineralogical composition of JSC Mars-1A, P-MRA, and S-MRA.

Mineral phase	JSC Mars-1A (wt%)	P-MRA (wt%)	S-MRA (wt%)
Plagioclase Feldspar (Ferric oxides)	64	–	–
Olivine	12	–	–
Magnetite	11	–	–
Pyroxene and/or glass	9	–	–
Fe_2_O_3_	–	5	–
Montmorillonite	–	45	–
Chamosite	–	20	–
Kaolinite	–	5	–
Siderite	–	5	–
Hydromagnesite	–	5	–
Quartz	–	10	3
Gabbro	–	3	31
Dunite	–	2	16
Hematite	5	–	17
Goethite	–	–	3
Gypsum	–	–	30

Composition as weight percent (wt%) of the mixture. Data for JSC Mars-1A were obtained from Morris et al. (1993); data for P-MRA and S-MRA were obtained from Dr. Jörg Fritz, Museum für Naturkunde Berlin, Germany.

**Table 2:** Major element composition of JSC Mars-1A, P-MRA, and S-MRA.

Major element composition	JSC Mars-1A (wt%)	P-MRA (wt%)	S-MRA (wt%)
Silicon dioxide (SiO_2_)	34.5–44	43.6	30.6–31.8
Titanium dioxide (TiO_2_)	3–4	0.36–0.45	0.05–0.98
Aluminum oxide (Al_2_O_3_)	18.5–23.5	11.2–11.9	5.6–9.2
Ferric oxide (Fe_2_O_3_)	9–12	19.6–20.3	14.9–19.9
Iron oxide (FeO)	2.5–3.5	–	–
Magnesium oxide (MgO)	2.5–3.5	4.48–4.52	10.3–10.9
Calcium oxide (CaO)	5–6	4.67–4.74	17.8–18.4
Sodium oxide (Na_2_O)	2–2.5	0.29–0.32	1.04–1.09
Potassium oxide (K_2_O)	0.5–0.6	1.04–1.07	0.13–0.86
Manganese oxide (MnO)	0.2–0.3	0.16–0.17	0.31–0.41
Diphosphorus pentoxide (P_2_O_5_)	0.7–0.9	0.55–0.56	0.05–0.42
Sulfur trioxide (SO_3_)	–	<0.1–0.2	2.7–9.1
Loss of ignition (LOI)	ND	11.8–12.4	5.4–6.4

Determined through x-ray fluorescence measurements. Data for JSC Mars-1A obtained from Orbital Technologies Corporation, Madison, WI, USA; data for P- and S-MRA obtained from Dr. Jörg Fritz, Museum für Naturkunde Berlin, Germany. For chemical composition the normal convention for data presentation uses oxide formulae from assumed oxidation states for each element and oxygen is calculated by stoichiometry (e.g., silicon is analyzed as an element but presented as SiO_2_). These are representations of the chemistry and do not represent actual phases or minerals in each simulant. ND = not determined.

The second and third MRAs, phylosilicatic MRA and sulfatic MRA (P- and S-MRA, respectively), were provided by the Museum für Naturkunde in Berlin and were produced by mixing terrestrial igneous rocks, phyllosilicates, carbonates, sulfates, and iron oxides obtained from KRANTZ (www.krantz-online.de). The minerals and rocks were chosen to be structurally and chemically similar to those identified in Martian meteorites (McSween, 1994) and on the surface of Mars (Bibring et al., 2005; Poulet et al., 2005; Chevrier and Mathé, 2007; Bishop et al., 2008; Morris et al., 2010). The components were mixed in relative proportions to obtain a mafic to ultramafic bulk chemical composition (**Tables 1** and **2**). The two different mineral and rock mixtures reflected the current knowledge of environmental changes on Mars: weathering or hydrothermal alteration of crustal rocks and the perception of secondary minerals during part of the Noachian and Hesperian epoch followed by the prevailing cold and dry oxidizing condition, with the formation of anhydrous iron oxides. The preparation of the two different mixtures account for the orbital observations that the phyllosilicate deposits are generally not occurring together with the sulfate deposits (Poulet et al., 2005).

Both mineral mixtures contain igneous rocks composed mainly of pyroxene, plagioclase (gabbro) and olivine (dunite). In addition to quartz, the anhydrous iron oxide hematite (α-Fe_2_O_3_), the only iron oxide that is thermodynamically stable under the present day Martian conditions (Gooding, 1978), was added to both mixtures. P-MRA resembles igneous rocks altered by pH-neutral hydrous fluids to clays of the smectite group, including montmorillonite, chamosite (Poulet et al., 2005) and the clay mineral kaolinite (Mustard et al., 2009). Siderite and hydromagnesite were included to account for carbonates that formed either by precipitation or interaction between a primitive CO_2_-rich atmosphere/hydrosphere and basaltic subsurface rocks (Chevrier and Mathé, 2007; Morris et al., 2010). S-MRA serves as an analog for a more acidic environment with sulfate deposits, and in addition to igneous rocks and anhydrous iron oxides, it includes goethite and gypsum. The materials were crushed to obtain a grain-size distribution for mechanically fragmented regolith, and to reduce nugget effects, only fragments <1 mm were used in the mineral mixtures. After mixing the different components, the size distributions of the mixtures were determined by sieving.

For all cultivation experiments with MRAs described here, the required amount of each MRA was weighed in serum bottles (125 ml and 25 ml). The bottles were then sealed with a butyl rubber stopper (thickness 12 mm) and an aluminum crimp, and anaerobic conditions were created by degassing (water-jet vacuum pump) and flushing with N_2_/CO_2_ (80:20 v/v) at 200 kPa. After autoclaving (121°C for 25 min), sterile medium or buffer solution prepared as described previously were added to the bottles.

### Influence of MRAs on the Activity of Methanogenic Archaea (First Experiment)

Microbial cells were grown to a cell density of 10^8^ cells ml^-1^, which is the late exponential phase, and 5 ml of the culture was transferred to 125-ml serum bottles containing 50 ml of fresh anaerobe medium mixed with the specific amount of MRA (0.0, 0.5, 1.0, 2.5, or 5.0 wt%). The starting cell concentration in the experimental serum bottles was approximately 5 × 10^7^ cells ml^-1^. The change in pH for samples containing 1.0 and 5.0 wt% MRA was measured separately, and all incubations and methane measurements were carried out as previously described.

### Growth of Methanogenic Archaea in Water-Saturated MRA Soils (Second Experiment)

**FIGURE 1:**
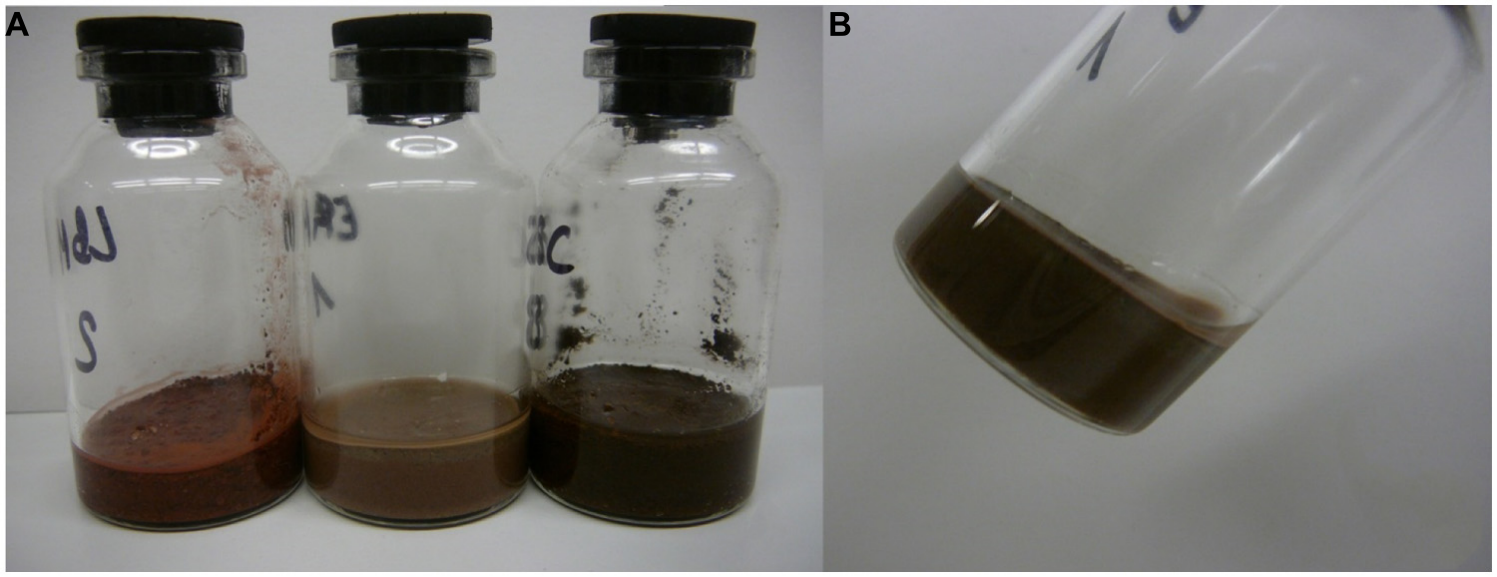
**Water-saturated Martian regolith analogs (MRAs) in 25 ml serum bottles.** Twenty-five milliliter serum bottles were filled with the different MRA to achieve an equal level of filling. Water was added until the water film line reached the surface of the MRA after 24 h of settling. **(A)** From left to right: S-MRA, P-MRA, and JSC Mars-1A. **(B)** Bottle with P-MRA held angular to display the water film at the glass margin.

To test for activity and growth of methanogenic archaea on MRA model soils, the strains were incubated on buffer-saturated MRAs containing NaHCO_3_ (4 g l^-1^), Na_2_S × 3H_2_O (0.3 g l^-1^) and resazurin (1 g l^-1^) as a redox indicator. The serum bottles used for this experiment had a volume of 25 ml. Due to the different densities and interstice volumes of the soil material, the total volume of buffer that was needed to achieve saturation differed for each MRA. Five grams of material and 3.1 ml of buffer were used for JSC Mars-1A, 8 g of material and 1.5 ml of buffer were used for S-MRA, and 5 g of material and 2.9 ml buffer were used for P-MRA. Examples of the test-bottles containing the three buffer-saturated MRAs are shown in **Figure 1**.

Cells were grown to a density on the order of 10^8^ cells ml^-1^, which is late exponential phase. To wash the cells, 50 ml of each growth culture medium was added to sealed screw-cap centrifuge tubes (Nalgene, VWR International, Germany; two parallel tubes were used for each methanogenic strain) and centrifuged at 4200 × *g* for 45 min. The supernatant was discarded, and the pellets were resuspended in buffer solution; this step was repeated twice. After the last centrifugation step, the cell pellets were resuspended either with buffer solution or with fresh medium (each 20 ml). One milliliter of each cell suspension was used as inoculum for each test serum bottle containing MRAs. Bottles with 4 ml of fresh medium and 1 ml of cell inoculum (resuspended in medium or buffer) were used as the positive controls, and the negative controls consisted of 4 ml of buffer with 1 ml of inoculum (cells resuspended in buffer). The resulting cell concentrations at the beginning of the experiment were approximately 4 × 10^7^ cells g^-1^ for all JSC Mars-1A and P-MRS samples, 5 × 10^7^ g^-1^ for the S-MRS samples, and 1.5 × 10^8^ ml^-1^ for the positive and negative control samples. Additional blank controls containing MRAs mixed with buffer or medium without cells were prepared to check for abiotic methane production. The bottles were incubated, and the methane production was measured as previously described.

### Tolerance of Methanogenic Archaea to Desiccation in the Presence of MRAs (Third Experiment)

In the third experiment, the effect of MRAs on the survival of desiccated methanogenic archaea was analyzed. Cells were grown as previously described but with 1 wt% of regolith added to the growth medium. No regolith was added to the control samples (desiccation on normal growth medium).

The strains were grown to a cell density of approximately 10^8^ cells ml^-1^ for all *Methanosarcina soligelidi* samples, 10^7^ cells ml^-1^ for all *Methanosarcina mazei* samples, and 10^9^ cells ml^-1^ for all *Methanobacterium movilense* samples. All cells were grown to the exponential or late exponential growth phase and were then harvested together with the Martian regolith analog (MRA) particles by centrifugation. Two 50-ml serum bottles of the growth media for each strain and sample condition (medium only, JSC Mars-1A, P-MRA, and S-MRA) were then transferred to centrifuge tubes (Nalgene, VWR International, Germany), sealed with a screw cap and centrifuged at 4200 ×*g* for 45 min at 4°C. After centrifugation, the tubes were placed in an anaerobic chamber, the supernatant was carefully discarded, and the cells as well as the cell-regolith pellets were resuspended in 1 ml (medium only), 4 ml (P-MRA and S-MRA), and 5 ml (JSC Mars-1A) of fresh medium. The cell suspensions were transferred to sterile 500-μl reaction tubes (Eppendorf, Germany) in aliquots of 20 μl (medium only), 80 μl (P-MRA and S-MRA), and 100 μl (JSC Mars-1A; these differences in volume were due to the different efficiency of the pipetting of the regolith-containing mixtures), which resulted in approximate total starting cell concentrations in the reaction tubes of 2 × 10^9^ (*M. mazei*), 2 × 10^10^ (*M. soligelidi*) and 2 × 10^11^ (*M. movilense*). The reaction tubes were then transferred to an anaerobic cylinder outside of the chamber and opened under a constant gas flow of N_2_/CO_2_ (80:20 v/v). The cylinder was subsequently sealed and flushed several times with H_2_/CO_2_ (80:20 v/v) through a valve system with sterile filters (0.2 μm), and the gas pressure inside the cylinder was adjusted to 1 bar overpressure to ensure anaerobic conditions. The cylinder was placed in the dark at room temperature (approximately 22°C), and 50 g of KÖSTROLITH^®^ (CWK, Chemiewerk Bad Köstritz GmbH, Bad Köstritz, Germany) was placed on the bottom of the cylinder to serve as a drying agent to desiccate the samples. Prior to use, the cylinder and drying agent were sterilized by UV irradiation for 1 h.

Depending on the sample type, no liquid phase was visible after 2–7 days of desiccation. At time intervals of 100, 200, 300, and 400 days, the samples were removed from the anaerobic container, and sampling was performed under a sterile gas flow of N_2_/CO_2_ (80:20 v/v). The reaction tubes were immediately closed before they were removed and directly transferred inside the anaerobic chamber.

To test the survival and activity of the desiccated cells, the samples were resuspended in fresh medium (200 μl) and left for approximately 6 h in the anaerobic chamber to allow the regolith to completely dissolve (samples from time step days 300 and 400 were left overnight). The resuspended samples were then mixed with 2 ml of fresh medium in a syringe and inoculated into sterile anaerobic, 5-ml serum bottles. After inoculation, the bottles were incubated, and methane production was measured as described earlier. The time intervals for the measurements ranged from 7 days (samples from time step 100) to 3 weeks (samples from time step day 200 and above), and incubation and measuring continued for up to 80 days after inoculation. All reincubation tests were performed in triplicate.

To estimate the number of cells with an intact cell membrane after the desiccation period, the samples were resuspended in a 1:1 mixture of diethyl dicarbonate water (DEPC) and fresh medium (200 μl in total). A volume of 0.5 μl of PMA (Biotium, Hayward, CA, USA) was added to the reaction tubes to a final concentration of 50 μM. After addition of PMA, which irreversibly binds to DNA of cells with damaged membranes and inactivates it for further processing (Taskin et al., 2011), the tubes were incubated for 5 min on a shaker inside an anaerobic chamber in the dark. The tubes were then placed on ice and irradiated with a 400 W halogen floodlight from a distance of 20 cm. During the 5 min of irradiation, the tubes were frequently shaken and rotated, and after irradiation, the DNA of the desiccated samples was extracted using an UltraClean^®^ Microbial DNA isolation kit according to the manufacturer’s instructions (MO BIO Laboratories, Inc., CA, USA). To increase the amount of eluted DNA, the last step was modified to two elutions with 25 μl of buffer each. Additionally, the elution buffer was warmed to 60°C before elution. The eluted DNA solution was kept frozen at –20°C until further processing, and isolated DNA from all samples was prepared in triplicate.

### Validation of PMA Treatment for Methanogenic Strains

To ensure that only DNA from intact cells was quantified, the PMA method in combination with quantitative PCR was tested separately. The three strains were grown as described previously. In two parallel approaches, cells were harvested by centrifugation (8800 ×*g* for 60 min) from 20 ml of each culture, and the cell pellets were resuspended in 5 ml of each medium, with one part of the samples treated with 70% isopropanol for 40 min to destroy the cell membranes, and the other part left untreated. After the isopropanol treatment, the samples were washed twice with fresh medium, centrifuged (10000 ×*g* for 30 and 15 min) and resuspended again in 5 ml of fresh medium. One milliliter of the treated and untreated samples was processed with PMA, as described above, or left unprocessed, respectively. The DNA was extracted from all samples, and quantitative PCR was performed to determine the gene copy numbers and hence the number of cells with intact membranes.

### Quantitative PCR

To estimate the number of viable cells after desiccation, the desiccated cell samples were treated with PMA as described previously. After isolation, the DNA was amplified by quantitative PCR (Rotor Gene Q Qiagen, Germany) using the methanogen-specific functional gene primer pair mlas-f and mcrA-r (Steinberg and Regan, 2008, 2009), which targets the alpha-subunit of methyl-coenzyme M reductase (*mcrA*). Based on the data currently deposited in the NCBI database, we assumed that each genome had a single copy of the *mcrA* gene; therefore, the gene copy numbers corresponded to the cell numbers.

The reaction mixture used for gene amplification included the following: 12.5 μl of SYBR green, 0.5 μl of each primer, 6.5 μl of DEPC water, and 5.0 μl of diluted template DNA (1:30). The PCR cycles were as follows: start, 95°C for 10 min; step 1, 95°C for 30 s; step 2, 55°C for 30 s; step 3, 72°C for 45 s; step 4, and 80°C for 3 s. Steps 1–4 were repeated 40 times. To acquire fluorescence data, the samples were melted from 50 to 95°C, with 5-s holding intervals, and the fluorescence data was acquired. The quantification of DNA was conducted using *Methanosarcina barkeri* as a standard at dilutions from 1.7 × 10^8^ to 1.7 × 10^4^ copies ml^-1^.

## Results

### Influence of Different MRAs on the Activity of Methanogens (First Experiment)

**FIGURE 2:**
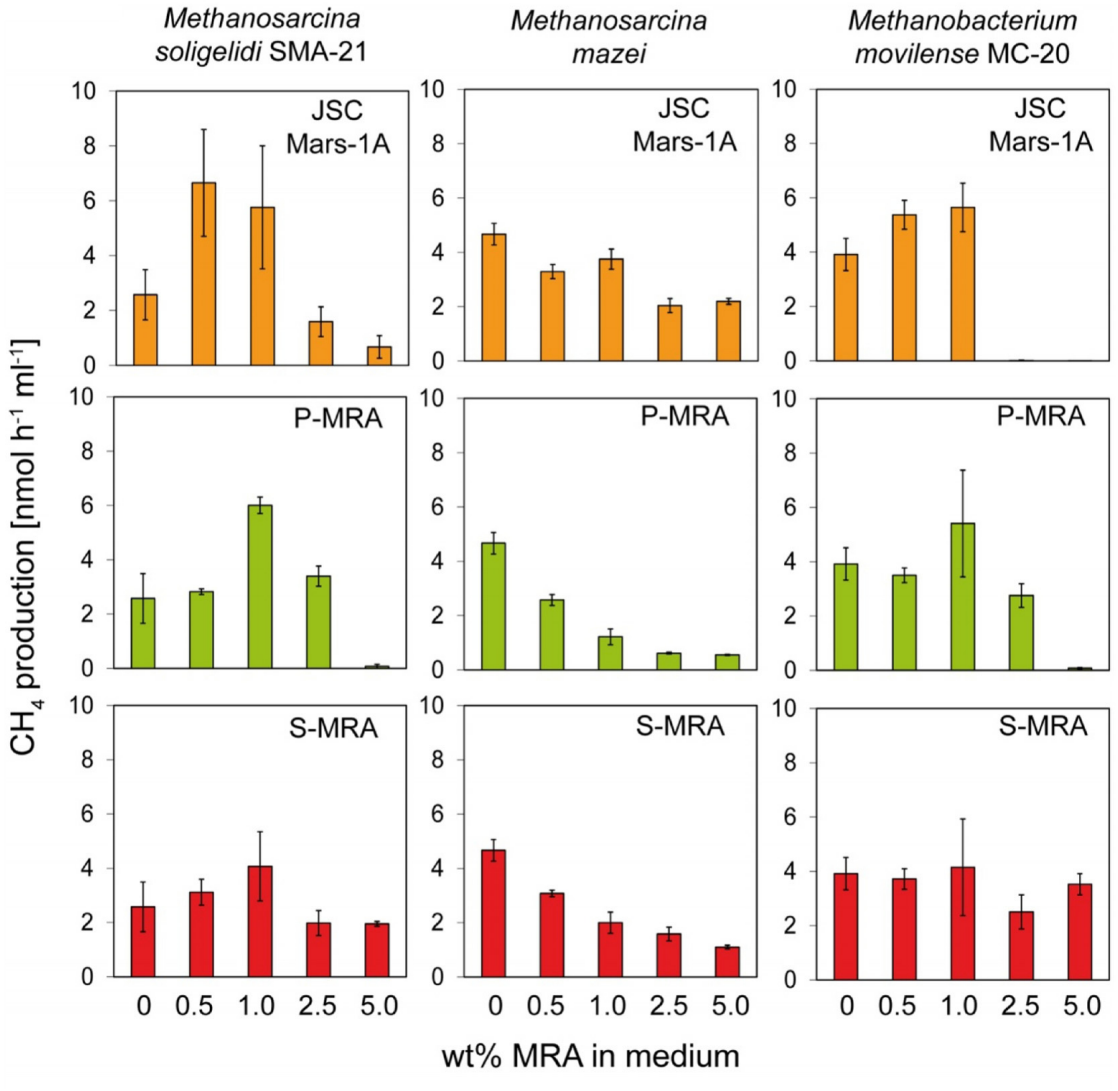
**Experiment 1, methane production rates of the methanogenic strains incubated with increasing concentrations of MRA.** The three methanogenic strains where incubated with increasing concentrations of the three MRAs added to the normal growth medium. The methane production rate was calculated from the increase of methane in the headspace. Error bars indicate SD, *n* = 3.

To determine the effect of the different MRAs on the metabolic activity of the archaea strains, we determined the methane production rates based on the linear increase in the methane concentrations measured after 8–10 days of incubation (**Figure 2**).

For all tested strains, MRA concentrations above 1.0 wt% resulted in decreased methane production rates. The methane production rate of *Methanosarcina soligelidi* was reduced from 2.6 ± 0.9 nmol CH_4_ h^-1^ml^-1^ without regolith to 0.7 ± 0.4 on 5 wt% JSC Mars-1A, 0.1 ± 0.1 on 5 wt% P-MRA and 1.9 ± 0.1 on 5 wt% S-MRA. The rates of *Methanosarcina mazei* were reduced from 4.7 ± 0.4 nmol CH_4_ h^-1^ml^-1^ on medium to 2.2 ± 0.1 (JSC Mars-1A), 0.6 ± 0.1 (P-MRA) and 1.1 ± 0.1 (S-MRA) when incubated with 5 wt% of the regoliths. The methane production rates of *Menthanobacterium movilense* were reduced from 3.9 ± 0.6 nmol CH_4_ h^-1^ml^-1^ to less than 0 (JSC Mars-1A), 0.1 ± 0.1 (P-MRA), and 3.5 ± 0.4 (S-MRA) when incubated on 5 wt% regolith; however, the latter was a negligible change compared to incubation on medium without MRAs.

Instead, at lower concentrations (0.5 and 1.0 wt%), MRAs had a positive effect on *M. soligelidi* and *M. movilense* and increased their methane production rates. The rates of *M. soligelidi* increased from 2.6 ± 0.9 nmol CH_4_ h^-1^ml^-1^ without regolith to 5.8 ± 2.2 (JSC Mars-1A), 6.0 ± 0.3 (P-MRA), and 4.1 ± 1.3 (S-MRA) with 1 wt% regolith. For *M. movilense,* the rates were from 3.9 ± 0.6 nmol CH_4_ h^-1^ml^-1^ on medium to 5.7 ± 0.9 (JSC Mars-1A), 5.4 ± 1.9 (P-MRA) and 4.2 ± 1.7 (S-MRA) on 1 wt% regolith. The methane production rates of *M. mazei* were reduced in the presence of regoliths in all experiments.

It has to be mentioned that incubation times longer than 40 days resulted in final concentrations of approximately 20% methane, which equaled the stoichiometric maximum concentration produced by the organisms when incubated under normal growth conditions. However, this methane concentration is usually achieved after fewer than 3 weeks of incubation. The only exception to this observation was *Methanobacterium movilense*, which produced up to 10% methane until day 50 when incubated in the presence of any concentration of MRA.

The changes in pH due to the addition of MRAs to the growth media were negligible. In general, the addition of JSC Mars-1A and P-MRA resulted in a slightly more basic pH, whereas the addition of S-MRA resulted in a more acidic pH.

### Growth of Methanogens in Water-Saturated MRAs (Second Experiment)

Methane production was measured by GC for up to 80 days. All positive controls showed continuous methane production, while the negative controls showed no methane production. The additional blank controls (MRA with medium or buffer) showed little methane production in some replicates, e.g., in S-MRA with buffer solution, where the concentration did not exceed 180 ppm after more than 80 days of incubation. All other tested MRAs reached approximately 30 ppm as a maximum value. To verify that this observed methane release was not due to biotic production through contamination, the blank control bottles were flushed again with fresh gas, and no further increase in methane could be measured.

**FIGURE 3:**
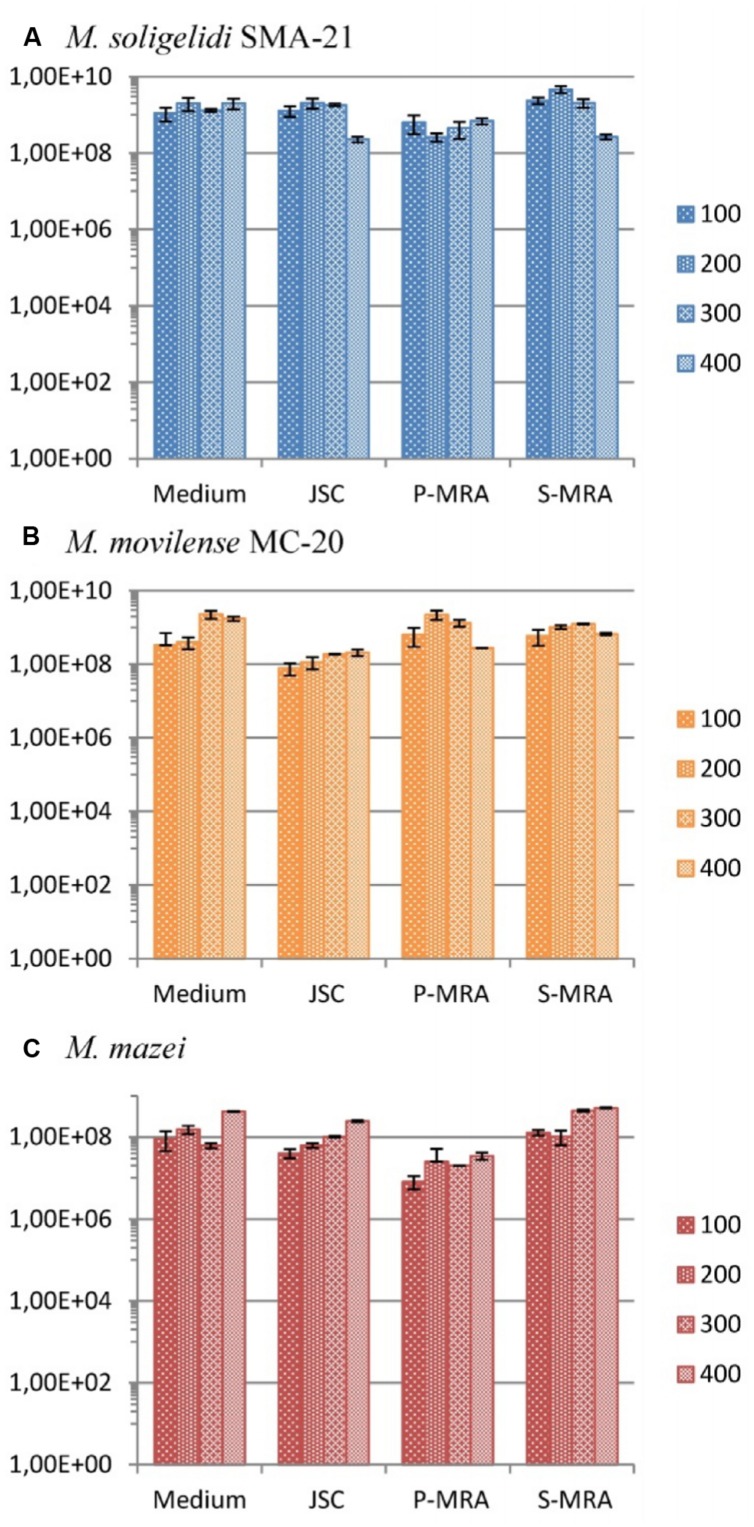
**Experiment 2, methane production of the three methanogenic strains over time when incubated on medium or buffer saturated MRA. (A,C,E)** Show the increase of methane over time for **(A)** *Methanosarcina soligelidi*, **(C)** *Methanosarcina mazei*, and **(E)** *Methanobacterium movilense* when incubated with standard growth medium on the three MRAs. Green line with closed triangles: P-MRA; red line with closed circles: S-MRA; orange line with closed squares: JSC Mars-1A. **(B,D,F)** Show the increase of methane over time for **(B)** *Methanosarcina soligelidi*, **(D)** *Methanosarcina mazei*, and **(F)** *Methanobacterium movilense* when incubated with buffer solution on the three MRAs. Green line with open triangles: P-MRA; red line with open circles: S-MRA; orange line with open squares: JSC Mars-1A; black line with crosses: highest production of blank controls without cells. All error bars indicate SD, *n* = 3.

The increases in the methane concentration during the incubation time for all combinations of methanogenic archaea, MRAs, growth media and buffer solutions are shown in **Figures 3A–F**. In general, all methanogenic strains were able to produce methane on at least one of the tested MRAs when incubated with both growth medium and buffer solution, although this production was lower on buffer than on growth medium. As shown in **Figures 3A,B**, *M. soligelidi* produced more than 20% methane on P-MRA and approximately 5% methane on S-MRA when incubated in medium, while it produced 0.3% methane on P-MRA when incubated in buffer. However, methane production did not exceed the concentration of the blank controls on S-MRA, and no methane was produced on JSC Mars-1A.

*Methanosarcina mazei* (**Figure 3C**) showed methane production of 8% only on S-MRA when incubated with medium, and it was able to produce methane on all three tested MRAs when incubated with buffer (**Figure 3D**). The final concentrations, 1.2, 0.9, and 0.4% methane (P-MRA, S-MRA, and JSC Mars-1A, respectively), were higher than that of the blank control.

*Methanobacterium movilense* produced more than 25% methane when incubated on P-MRA with medium and 9.4% methane when incubated on S-MRA with medium (**Figure 3E**). Incubation with buffer resulted in a concentration of more the 20% on P-MRA and of 1.7% on S-MRA. *M. movilense* did not produce methane on JSC Mars-1A.

### Growth of Methanogens after Desiccation on MRAs (Third Experiment)

Reincubation of the desiccated cell samples showed that methane production could be measured even after 400 days of desiccation, and all strains were able to survive the complete desiccation period under at least three of the four tested conditions. **Table 3** shows the results of methane production after incubation for 80 days. For a better comparison, the produced methane concentrations were rated at levels 0 to 3 on analog to heat map charts. Level 0 indicated no detected methane or that the measured concentration was below 20 ppm; level 1 indicated a methane concentration above 20 but below 100 ppm; level 2 indicated methane concentrations between 100 and 10,000 ppm; and level 3 exceeded 10,000 ppm (1%). The most important factor for identifying actual methane production was a constant increase in the methane concentration over time, even for the samples marked “1,” whose final concentration of methane did not exceed 100 ppm.

**Table 3:** Rated methane production after the specific time steps of desiccation measured for up to 80 days of reincubation.

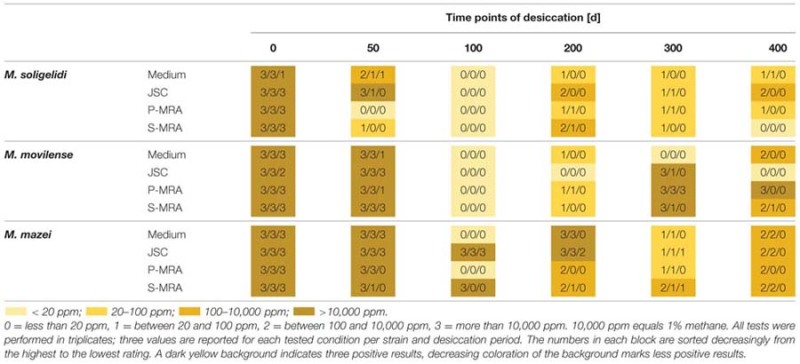

Reincubation of the desiccated samples showed the most constant results for *M. mazei*. In this case, the highest numbers of the tested triplicates were producing methane at least on levels 1 and 2. The highest measured methane production after 400 days of desiccation was detected for *M. movilense* when desiccated on P-MRA, while the weakest results were observed for the time point day 100, on which none of the *M. soligelidi* or *M. movilense* samples showed any methane production. For further verification of methane production, the samples of the last two time points (day 300 and 400) were flushed with a fresh gas mixture (N_2_/CO_2_, 80:20 v/v) after the first series of measurements (80 days) and incubated again because the headspace pressure in the serum bottles might have dropped due to repeated sampling. Within a few weeks, most samples showed the same level of methane production that was measured at the beginning of the experiments; however, some of the samples did not produce methane. This was the case for some level 1 production from time point day 300 for all strains (*M. soligelidi* on medium, JSC Mars-1A, and P-MRA; *M. movilense* and *M. mazei* on JSC Mars-1A and S-MRA) and for one level 1 production of *M. movilense* on S-MRA at time point day 400. In contrast, two of the level 1 productions at day 300 for *M. mazei* (on JSC Mars-1A and S-MRA, respectively) turned out to be level 3 and level 2 productions when incubated after flushing of the headspace.

### Validation and Application of PMA Treatment in Combination with qPCR

When combined with PMA treatment, qPCR is a valid method to estimate the number of cells (with intact membranes) based on the DNA copy numbers (Taskin et al., 2011). A clear difference was observed in the copy number estimation for the samples treated with isopropanol, depending on whether PMA was added before the DNA isolation. At best, 0.2% of the copy numbers of the samples not treated with PMA could be found in the PMA samples. For the samples not treated with isopropanol, a difference in the detected copy numbers could also be observed, and treatment with PMA before DNA isolation resulted in reduced copy numbers. At a minimum only approximately 10% of the copy numbers of the untreated sample could be found in the PMA sample. This was the case for *M. soligelidi*, and the other two strains had approximately 70% (*M. movilense*) and 30% (*M. mazei*) of the copy numbers of the untreated samples.

**FIGURE 4:**
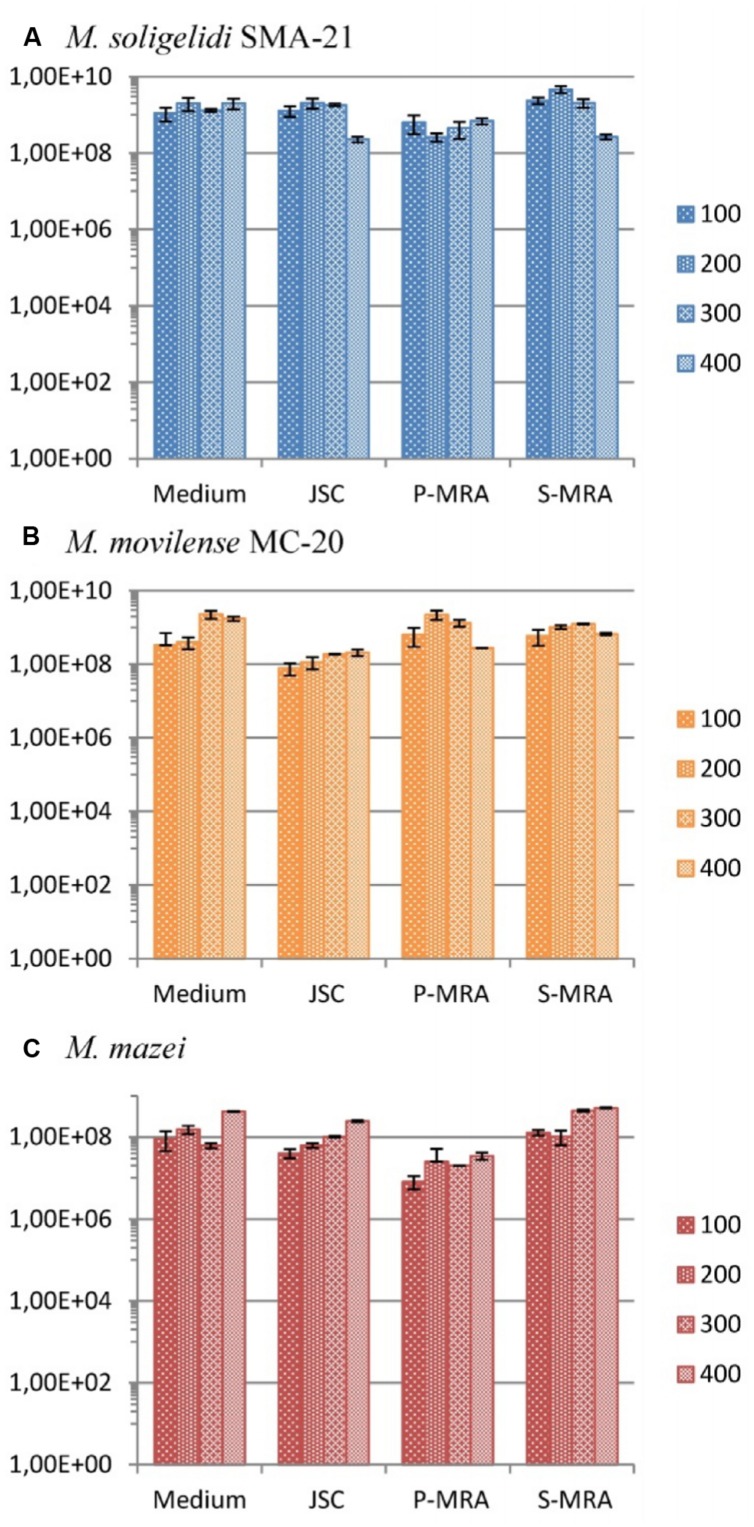
**Gene copy numbers (*mcrA*) per ml of culture medium during the desiccation period (primers mlas-f and mcrA-r). (A)** *Methanosarcina soligelidi*, **(B)** *Methanobacterium movilense*, **(C)** *Methanosarcina mazei*. All tests performed at least in triplicates, error bars indicate SD. The reaction efficiencies for all amplification runs were 84 ± 3 %, *R*^2^-values were 0.9973 ± 0.031.

The calculated gene copy numbers per milliliter of culture medium during the desiccation period are shown in **Figure 4**. Although there were variations in the estimated cell concentrations, in most cases, the gene copy numbers did not significantly change, as it was seen with a student’s *t*-test analysis for most of the tested conditions. Moreover, the variations were in the range of the SD. A high concentration of intact cells for all three methanogenic strains at all four conditions and even after 400 days of desiccation was detected (**Figure 4**).

## Discussion

Due to their ability for chemolithoautotrophic and anaerobic growth and their evolutionary origin in a time when global environmental conditions on Mars and Earth were supposedly similar (Carr, 1989, 1996; Durham et al., 1989; McKay and Davis, 1991; McKay et al., 1992), methanogenic archaea are considered ideal model organisms for studying possible life on Mars (Boston et al., 1992; Jakosky et al., 2003; Kral et al., 2004; Krasnopolsky et al., 2004; Morozova and Wagner, 2007; Morozova et al., 2007; Kral et al., 2011; Schirmack et al., 2014a). In this study, we investigated the effect of different Martian regolith analogs (MRAs) on the metabolic activity and desiccation resistance of methanogenic archaea. Our results prove that the tested methanogenic species have a long-term desiccation resistance (of more than 400 days) and are able to produce methane when incubated on a buffer solution and with MRAs alone.

The methane production rates of the strains *Methanosarcina soligelidi* and *Methanobacterium movilense* increased in the presence of MRAs up to a concentration of 1 wt%. It was noted that each species was differentially affected by the addition of the regoliths. A possible explanation for these differences may be related to the different habitats in which the strains were originally isolated and therefore their specifically adapted physiology. *M. movilense,* for example, inhabits H_2_S-rich groundwater (Sarbu et al., 1996), which could explain its higher tolerance to the sulfur-rich S-MRA. In general, the addition of regolith had, up to a certain level, a positive effect on methane production, likely by providing important trace elements such as nickel, cobalt and zinc, which are necessary for the metabolism of the organisms. Additionally, cells attached to regolith particles might have benefited from a shielding effect against environmental influences (Wagner et al., 1999). These positive effects might have become less important with increasing concentrations of MRA in the growth media, and thus, the activity of the methanogens may have been reduced due to inhibitory effects of the mineral mixtures, such as increasing sulfur concentrations. A comparable observation was made by Kral and Altheide (2013), who showed that the activity of methanogens was decreased in the presence of different Mars analog minerals such as the commonly used JSC Mars-1.

In the second experiment using buffer-saturated Martian regolith analogs, all tested methanogenic strains were able to produce methane in the presence of at least one regolith without any additional nutrients. However, the highest methane production was achieved for all strains after incubation on P-MRA. The production of methane alone might not be proof for actual growth, but in the case of *M. movilense,* which reached a final methane concentration of more than 20% when incubated on P-MRA, it can be assumed that growth related to high metabolic activity took place. This is in accordance with the study of Kral et al. (2004), which showed growth of *Methanothermobacter wolfeii* under comparable conditions on JSC-Mars 1, which was quite similar to the JSC Mars-1A tested here. Nevertheless, a buffer solution and a source of energy and carbon (H_2_/CO_2_ provided in the headspace) alone are not sufficient to support methanogenic activity, as no methane production could be observed in the control samples containing buffer and cells alone. If *M. movilense* has grown when incubated on P-MRS, the used mineral mixture (**Table 2**) could be a possible source of phosphorous. Nitrogen is present as molecular nitrogen in the headspace, which can be used by at least some strains of methanogenic archaea such as *Methanosarcina barkeri* (Murray and Zinder, 1984; Leigh, 2000) and *Methanobacterium bryantii* (Belay et al., 1988; Leigh, 2000), which belong to the same genus as *M. movilense*. So, in theory, *M. movilense* might be able to grow diazotrophically; however, this would of course need further verification.

It is remarkable that all of the tested strains were able to sustain the different conditions during the third experiment with up to 400 days of long-term desiccation. For the desiccation test, the quantified gene copy numbers of samples grown on medium only did not change significantly over the course of the experiment. Due to the PMA (DNA intercalating dye) treatment before DNA isolation and qPCR, damaged cells or free DNA were excluded from the quantification of the *mcrA* genes due to the formation of PMA-DNA complexes. This effect was shown, for example, by the study of Taskin et al. (2011), which tested this method on *Escherichia coli*. The results of the PMA validation experiment also demonstrate the effectiveness of this method for methanogenic archaea. For the control samples treated with isopropanol to destroy cell membranes, almost no DNA could be quantified when processed with PMA prior to DNA extraction. The lower copy numbers of the samples processed with PMA compared to the unprocessed samples showed cells with damaged membranes, where PMA could penetrate, in every culture. However, it is known that cell wall integrity also depends on the growth phase of the culture (Pagán and Mackey, 2000). Moreover, a portion of the intact cells might also have been destroyed during the handling of the samples before the PMA was inactivated by light, and therefore, they were not detected by qPCR. It is notable that the cells maintained their cell wall integrity when desiccated on medium. There was no indication of a positive effect of the added MRAs on cell wall integrity in any of the qPCR experiments, whereas a slightly negative trend was observed in some cases. Therefore, the desiccation resistance of the tested organisms can not be only related to shielding effects of the regolith particles. Other possible reasons could be the secretion of extracellular polysaccharides (EPS) that act as a protective layer, as was shown for *Methanosarcina barkeri* in the study of Anderson et al. (2012). In that study, EPS increased the resistance of the strain against desiccation as well as against other environmental stresses, such as oxygen exposure (for 7 days) and high temperature (up to 100°C).

Due to the application of PMA treatment followed by qPCR, it is possible that a large part of the cells was still intact and viable and therefore survived the desiccation period, even if the methane production that was detected after reincubation of the desiccated samples was comparatively low, which might be dependent on a prolonged lag phase. It is also possible that a portion of the organisms were in a dormant state and therefore not active or just active at a much reduced rate (Hoehler and Jørgensen, 2013). However, in the case of *M. movilense* desiccated for 400 days on P-MRA, the highest production of methane was detected after rehydration. The reason why some of the samples at early time points (e.g., at time step day 100, **Table 3**) showed no methane production, but samples at later stages did, cannot definitively be answered. A possible explanation may be the biological variability of the desiccation resistance and activity of the cells. It is also possible that, although the preparations were properly mixed, the samples were not entirely homogeneous. In addition, two of the samples on the starting date showed only little methane production, whereas all other samples reached several percentages of methane production.

Considering all of the results of the experiments, a phyllosilicate-rich soil environment seems to provide the best mineral mixture for methanogenic activity and survival under Mars analog conditions. The major difference between the mineral composition of P-MRA and those of JSC Mars-1A and S-MRA is its high content of phyllosilicate montmorillonite (clay mineral), which is known for its water-binding capacity and expansiveness when exposed to water. This characteristic may be one reason for the resistance of the cells to long-term desiccation in the presence of this mineral. Thus, these cells might have a sufficient source of water present during the desiccating conditions – at least for the later time period when compared to the other MRAs with less clay mineral content. Furthermore, montmorillonite also increases the ion-exchange capacity of P-MRA, which might be a major factor for the increased activity of methanogens on this MRA. Interestingly, montmorillonite has also been discussed as a positive factor influencing the formation of primitive lipid cells or cell precursors as well as RNA binding and therefore is hypothetically involved in the origin of life (Hanczyc et al., 2003).

## Conclusion

In the scope of the habitability of Mars, it is important for organisms to find all nutrients necessary for growth as well as sources of energy and carbon. Our experiments have shown that, besides being provided hydrogen and carbon dioxide, which are present in the Martian environment, the mineral mixtures of the MRAs contain all relevant nutrients to enable metabolic activity of methanogenic archaea. Albeit survival in a diurnal variation of simulated Martian surface analog conditions for 3 weeks was proven for *M. soligelidi* (Morozova et al., 2007), the surface-near or deeper subsurface would be more likely habitats due to the relatively stable thermo-physical conditions and better protection from radiation (Jones et al., 2011) as well as better access to liquid water and energy (Vance et al., 2007; Ulrich et al., 2012; Michalski et al., 2013). *M. soligelidi* has also shown its potential for active metabolism under Mars subsurface analog conditions (Schirmack et al., 2014a). In the previous study by Morozova et al. (2007), *M. soligelidi* exhibited an explicitly higher survival rate after 3 weeks of exposure to simulated Martian surface analog thermo-physical conditions compared to *M. movilense*. With regard to the results from the present study, it seems that *M. movilense* might be better adapted to cope with the single stress factor of desiccation in the presence of MRAs. *M. movilense* was isolated from the Movile cave, which is the first terrestrial ecosystem based on chemosynthesis (Sarbu et al., 1996) and therefore can be regarded as an analog for extraterrestrial subsurface habitats. The findings of this study may be valuable for future life detection missions, for example ExoMars, which is planned for 2018 and will sample the Martian subsurface (Baglioni and the EXM Rover Team, 2013).

## Conflict of Interest Statement

The authors declare that the research was conducted in the absence of any commercial or financial relationships that could be construed as a potential conflict of interest.
